# Identification of relevant analytical methods for adeno-associated virus stability assessment during formulation development

**DOI:** 10.1128/spectrum.01806-24

**Published:** 2025-06-30

**Authors:** Carina Rodenstein, Eva Schmid, Natalia Markova, Andreas Seidl

**Affiliations:** 1 Leukocare AG 722141 , Martinsried, Germany; 2 Malvern Panalytical Ltd. 36566 , Malvern, Worcestershire, United Kingdom; University of Miami, Miami, Florida, USA

**Keywords:** gene delivery, adeno-associated virus, formulation, stability, forced conditions, differential scanning calorimetry, fluorescence spectroscopy, light scattering (dynamic/static), HPLC

## Abstract

Formulation development for gene therapy viral vectors requires high-performance analytical methods that indicate or predict virus stability. Additionally, if testing involves multiple excipients, sample throughput must be sufficiently high, and the method should require low amounts of virus material. These are challenging and sometimes conflicting requirements, especially in the analysis of adeno-associated viral vectors, which are currently the most prominent viral vectors in gene therapy development. In this exploratory study, we evaluated the ability of different analytical methods to indicate or predict the stability of the adeno-associated viral vectors and identified highly suitable techniques that fulfilled the requirements for analysis during formulation development. As functionality assays, such as reporter gene assays, are already established for rapid quality control assessments, they are not addressed in this study. From this study, we conclude that two methods have great potential to predict the stability of adeno-associated viral vectors: extrinsic differential scanning fluorimetry using SYBR Gold as a DNA binding fluorescent dye to detect genome release and nano differential scanning fluorimetry to investigate capsid unfolding behavior (based on intrinsic protein fluorescence). Size-exclusion chromatography, using multi-angle light scattering and UV detection, was found to be stability-indicating for particle size distribution and determining genome load of adeno-associated viral vectors.

**IMPORTANCE:**

Viral vectors are of growing importance in the areas of gene therapy, oncology, and vaccine development. However, these vectors are very unstable and usually have to be stored frozen at very low temperatures due to sub-optimal formulation conditions, in many cases. The development of superior formulations for viral vectors requires high-performance analytical methods. In this study, we evaluated relevant analytical methods with respect to sample throughput, material consumption, and applicability for viral vector formulation development. To our knowledge, this is the first time that methods for viral vector analysis were categorized according to their power to predict or indicate time-dependent long-term stability. This categorization of analytical methods is essential to rationalize, accelerate, and enhance the formulation development of viral vectors. Therefore, the studies in this article are prerequisites for the development of more stable viral vectors for gene therapy and vaccines and higher yields in manufacturing.

## INTRODUCTION

Recombinant adeno-associated viruses (AAVs, for abbreviations, see Table 1) are a class of non-enveloped viral vectors that are being intensively investigated in the development of gene therapies due to their particular characteristics. They are considered non-pathogenic, deliver genes to many cell types, and ensure long-term expression in post-mitotic cells (1, 2). In order to develop efficient AAV therapies, multiple challenges need to be addressed, starting with the capsid design and the subsequent combination of a production and purification process that achieves high virus yields and full-empty capsid ratios. Moreover, optimal formulation conditions must be identified that reduce the virus’s vulnerability to different kinds of external physical stress factors during storage, handling, and application. To identify these, extensive characterization (capsid count, percentage of full capsids, particle size, potential aggregate formation, capsid stability, and infectivity) of the AAV samples is required.

**TABLE 1:** Abbreviations

Abbreviation	Description
AAV	Adeno-associated virus
Ad	Adenovirus
AEX	Anion-exchange chromatography
AUC	Analytical ultracentrifugation
ddPCR	Droplet digital polymerase chain reaction
DLS	Dynamic light scattering
DSC	Differential scanning calorimetry
DSF	Differential scanning fluorimetry
eDSF	Extrinsic differential scanning fluorimetry
HEK	Human embryonic kidney
HPLC	High-performance liquid chromatography
HRP	Horseradish peroxidase
IU/mL	Infectious units per milliliter
LFC	Laser force cytology
MADLS	Multi-angle dynamic light scattering
MALS	Multi-angle light scattering
MVA	Modified-vaccinia Ankara virus
NanoDSF	Nano differential scanning fluorimetry
NC	Negative control
NTA	Nanoparticle tracking analysis
qPCR	Quantitative polymerase chain reaction
Ref	Reference buffer (PBS, 0.001% poloxamer 188)
SEC	Size-exclusion chromatography
SLS	Static light scattering
UV	Ultraviolet
vg/mL	Viral genome per milliliter

Several techniques have been described to analyze these AAV attributes, e.g., droplet digital polymerase chain reaction (ddPCR), analytical ultra centrifugation (AUC), dynamic light scattering (DLS), differential scanning calorimetry (DSC), size exclusion chromatography- multi-angle light scattering (SEC-MALS), and differential scanning fluorimetry (DSF) (1, 3–6). However, it is still a challenge to identify analytical methods that fulfill critical method attributes, including low virus material consumption, high throughput, and the ability to indicate or predict virus stability. Such methods could act as a surrogate for the performance of laborious and resource-intensive functionality assays, at least in early phases of formulation development, where operators face a high number of samples and diversity in compositions. Especially, techniques predicting virus stability are of high interest during formulation development, as they might reduce the need for time-dependent stability studies, which are time-, material-, and cost-intensive. Functionality assays, such as reporter gene assays, are already established for rapid quality control assessments but are not addressed in this study (7).

**Fig 1:**
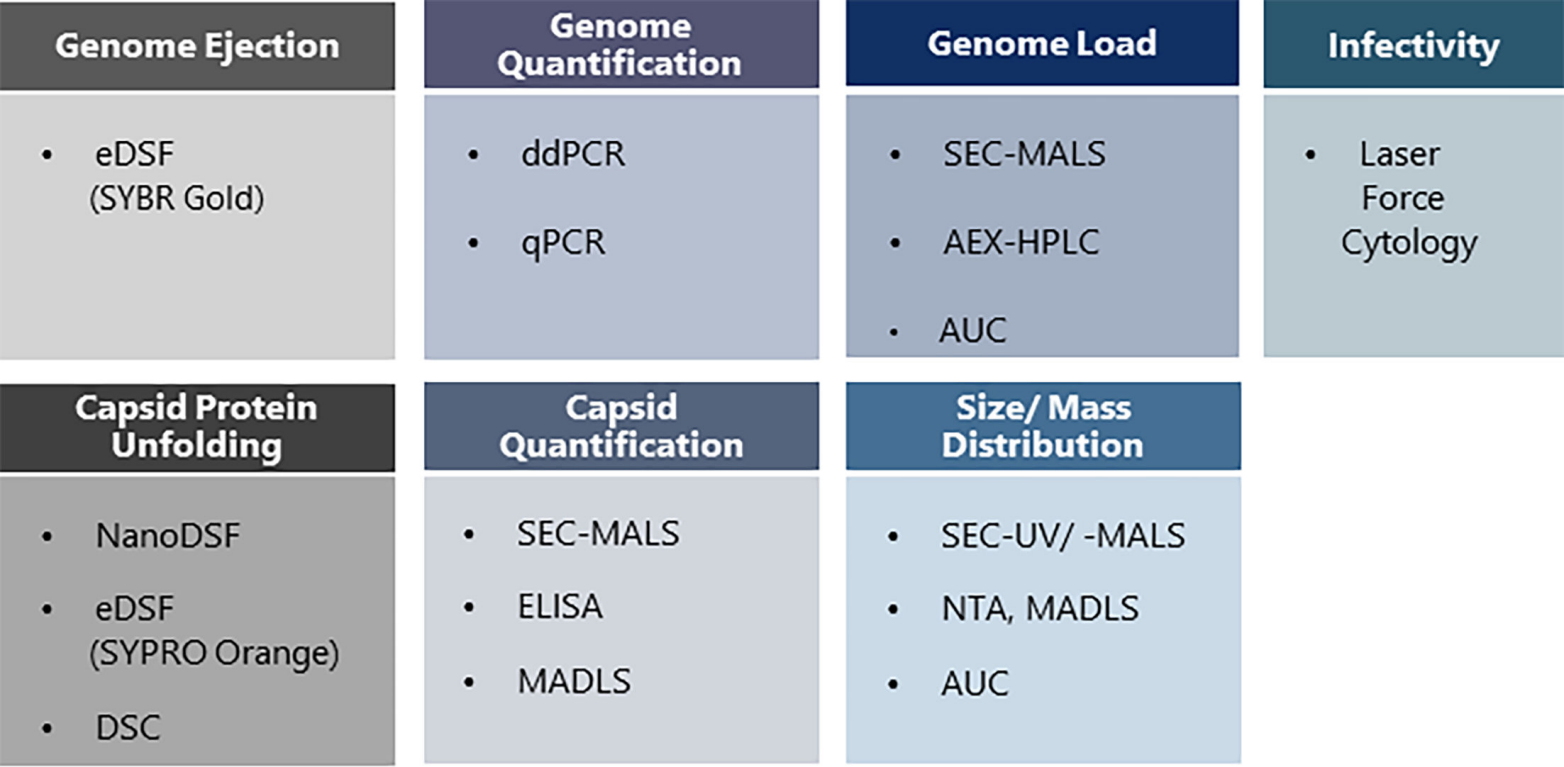
Analytical methods evaluated in this study.

In this study, we address the need for a better understanding of analytical methods using AAV serotype 2 and 5 (AAV-2 and AAV-5) with the insert Factor IX as a model with realistic genome size and compare current state-of-the-art analytical techniques that study different virus attributes (Fig. 1). The use of AAV-2 and AAV-5 showing differential susceptibility toward degradation facilitated the discrimination of analytical methods according to their stability-indicating and predictive properties. For this purpose, AAV-2 and AAV-5 samples in standard storage buffer (PBS/0.001% poloxamer 188; reference buffer) and several designed formulation buffers were compared before and after accelerated aging. The designed formulation buffers used in this study were selected based on a previous internal study in which they demonstrated different potential to stabilize AAVs in regard to capsid concentration, genome titer, and prevention of aggregation. Stability-indicating methods describe the stability of a drug substance over time when stored at the intended storage conditions or conditions for accelerated aging, whereas methods predicting stability predict at timepoint *t* = 0, the time-dependent stability of the drug substance (without applying time-dependent stress).

## RESULTS

### Particle size distribution analyzed by DLS, MADLS, and SEC-UV

Particle size distribution was studied by size-exclusion chromatography using UV detection (SEC-UV), dynamic light scattering, and multi-angle DLS (MADLS) (Fig. 1). A comparative evaluation of the techniques’ consumption of virus material with the respective instruments, the possibility of high-throughput screening, and the amount of lab work associated with these techniques suggests that SEC-UV is the most suitable method for investigating and comparing the stability of various AAV samples (Table 2).

**TABLE 2:** Amount of AAV material and lab work required for the performance of the tested analytical methods

Analytical methods tested in this study	Minimum AAV material required/measurement			High-throughput possibility	Amount of lab work^*a*^
	Amount (µL)	Concentration unit			
NanoDSF	25	3.00E + 12	cp/mL	Yes	+
eDSF (SYPRO Orange)	35	4.00E + 12	cp/mL	Yes	+
eDSF (SYBR Gold)	8	8.00E + 11	vg/mL	Yes	+
DSC	350	6.00E + 12	cp/mL	No	++
DLS	20	1E + 12-5E + 12	cp/mL	Yes	+
MADLS	50	1E + 12-5E + 12	cp/mL	No	+
AUC	400	1E + 12-5E + 12	cp/mL	No	++
SEC-UV	35	8.00E + 11	cp/mL	Yes	++
AEX-HPLC	25	1.00E + 12	cp/mL	Yes	++

^*a*^ ++ indicates that for this method more lab work is required in comparison for methods labeled with +.

In order to evaluate the techniques’ potential to indicate or predict AAV stability, AAV-5 in reference buffer and two designed formulations (F01 and F02) were compared. The samples were analyzed before and after the application of time-dependent stress conditions.

**Fig 2:**
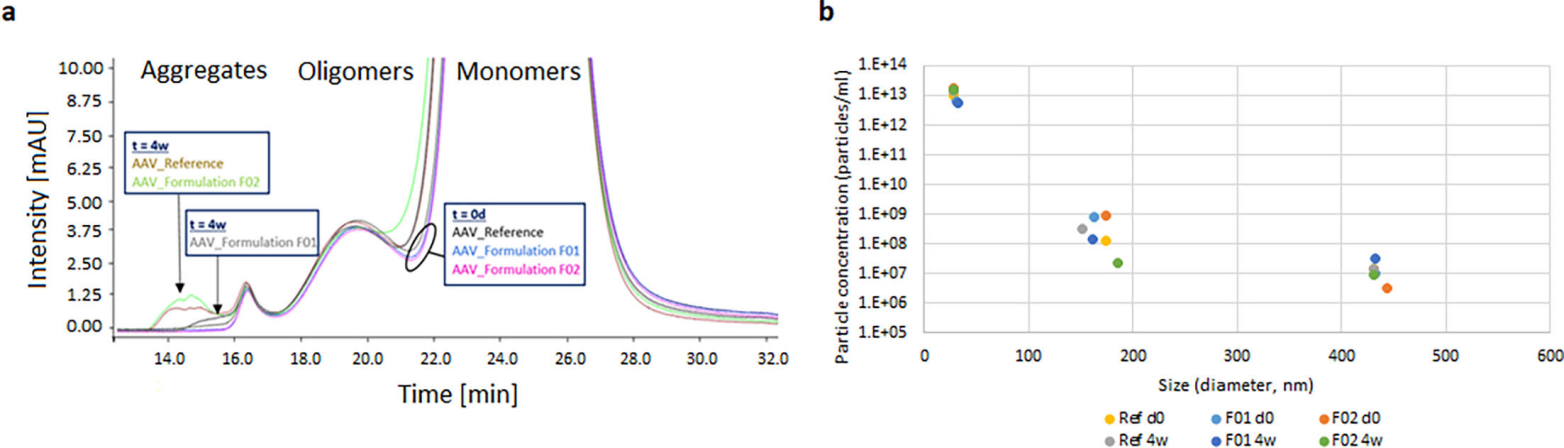
SEC-UV at 280 nm (a) and MADLS (b) results of AAV-5 in reference buffer and formulation F01 and F02. For both techniques (a and b), the results of the measurement before (*t* = 0 days) and after (*t* = 4 weeks) thermal stress at 40°C for 4 weeks are depicted (*n* = 1). The arrows show additional aggregate formation for specific AAV-5 samples. For MADLS, encircled are the three detected populations of specific size.

SEC-UV and MADLS with non-stressed AAV-5 samples (*t* = 0 days) revealed three particle populations for AAV-5 in all three buffers (Fig. 2). With both techniques, the monomeric virus particles formed the majority of the detected particles. Oligomers and aggregates were less present. SEC-UV data were highly consistent between the three non-stressed samples, as seen by the overlay of the AAV peak profile (Fig. 2a). MADLS, on the other hand, revealed slight variance between the three non-stressed formulated samples (Fig. 2b), as indicated by different concentrations and sizes measured for monomeric virus particles. In addition, SEC-UV revealed a very low SD in size and quantity of the particles for measurement replicates, indicating that this technique is very precise, and even small differences between formulated AAV samples can be detected.

Using the data obtained from the non-stressed AAV samples, it was further evaluated whether the techniques are able to indicate the stabilizing effect of formulations on AAV upon application of a stress condition. Accordingly, data derived from AAV-5 samples in three formulations subjected to thermal stress within an accelerated aging study (40°C for 4 weeks) were compared. With SEC-UV, an additional aggregate peak of different size and retention time was detected for AAV-5 in the three formulations after 4 weeks, indicating increased particle heterogeneity after thermal stress application (Fig. 2a). With MADLS, on the other hand, we saw no considerable differences between the formulated AAV-5 samples before and after thermal stress application (Fig. 2b). The reason for this difference between SEC-UV and MADLS might be the application of the adaptive correlation algorithm of the MADLS system, which suppresses very low-abundance transient scatters, such as dust and other contaminants, but may reduce the sensitivity for low-abundance aggregates.

**Fig 3:**
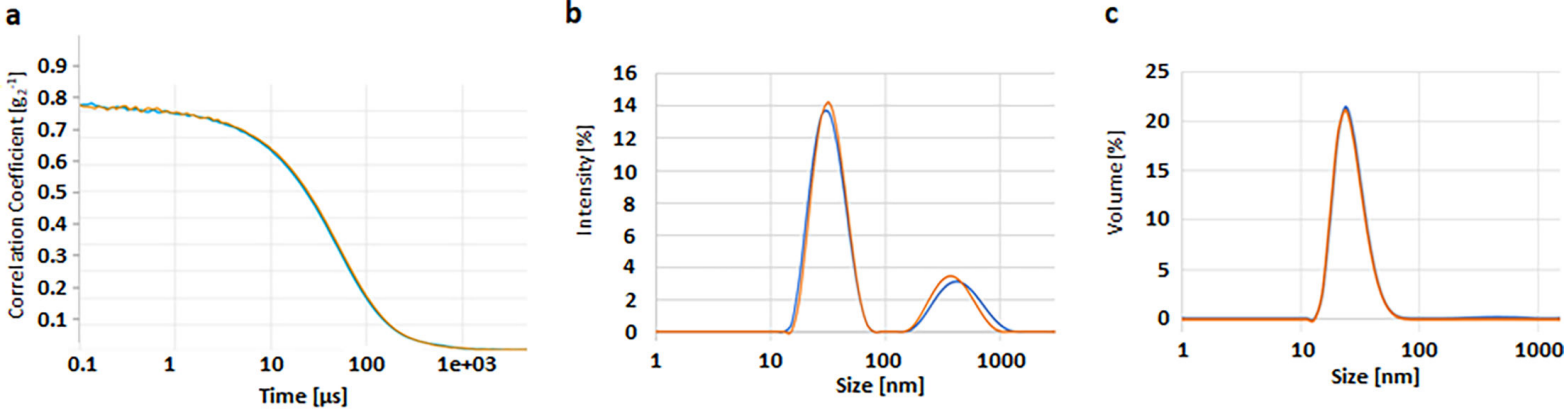
Isothermal DLS results of AAV-5 in formulation F02 before (*t* = 0 days, blue) and after (*t* = 4 weeks, orange) thermal stress at 40°C for 4 weeks. Shown are the correlation curve (a) and the size distribution blotted against signal intensity (**b**) and volume percent (**c**).

The alternative technique of isothermal DLS of AAV-5 in the formulation F02 revealed only two particle populations in an intensity-weighted readout with a main peak containing particles of 20–90 nm and a second peak with particles >100 nm (Fig. 3b). The evaluation of the polydisperse samples by volume, however, demonstrated that the particles >100 nm were only very few in number (Fig. 3c).

When comparing the stressed and non-stressed AAV-5 for formulation F02 tested, it could be seen, e.g., by the overlays, that the difference between stressed and corresponding non-stressed samples was marginal for intensity-weighted and volume-weighted particle size distribution as well as for the correlation function (Fig. 3a through c).

**Fig 4:**
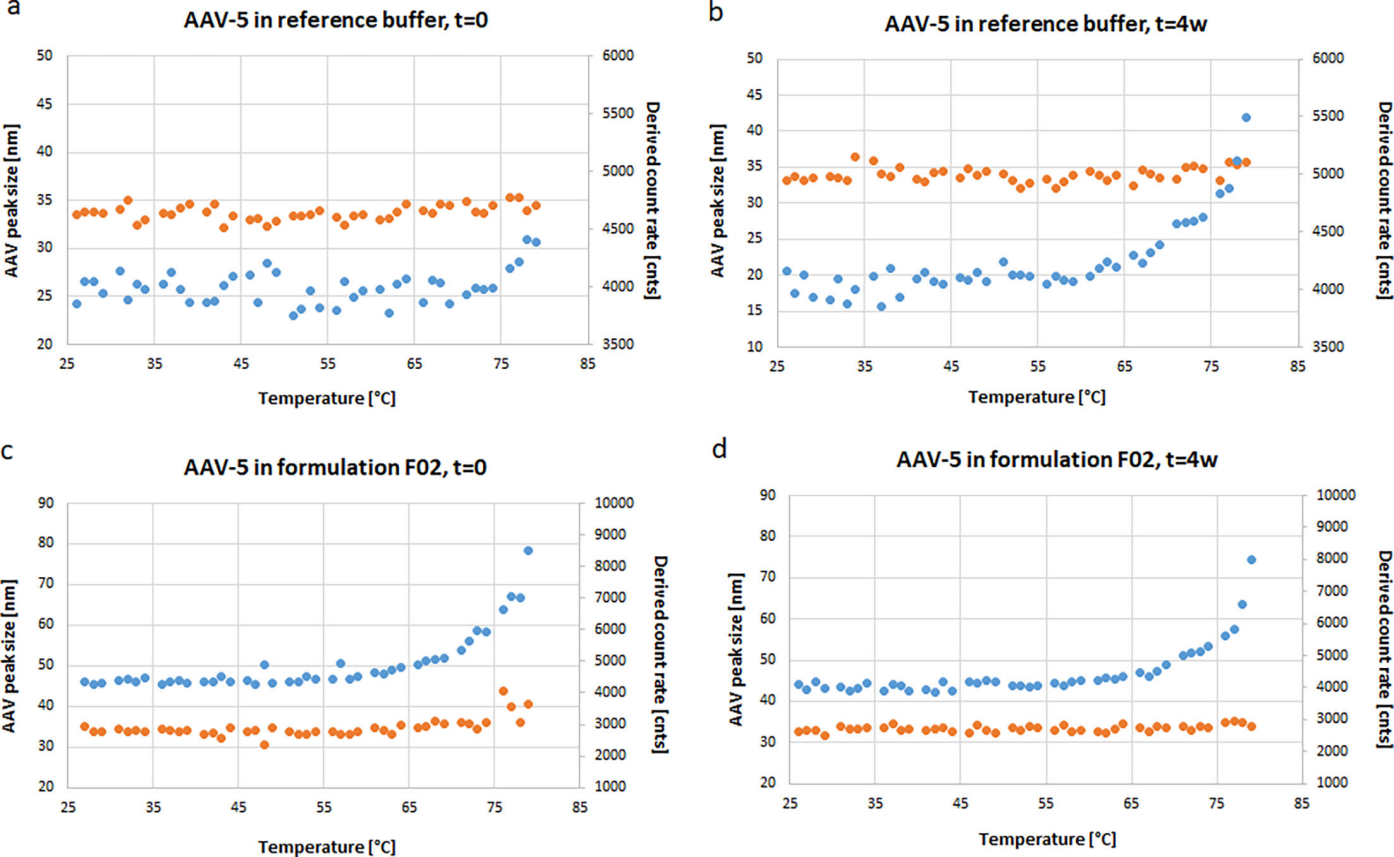
DLS results of AAV-5 in the reference buffer and formulation F02 during thermal ramp application. Depicted are the samples from the accelerated aging study, AAV-5 in reference buffer before (**a**, *t* = 0 days) and after (**b**, *t* = 4 weeks) storage at 40°C for 4 weeks, and AAV-5 in formulation F02 before (**c,** *t* = 0 days) and after (**d**, *t* = 4 weeks) storage at 40°C for 4 weeks. Orange, AAV-monomer peak size; blue, overall scattering intensity is given in derived count rate.

The addition of a thermal ramp during DLS measurement induced small differences in the AAV-5 size distribution regarding size and scattering intensity when determined by DLS (Fig. 4) as compared with AAV-5 in the reference buffer and formulation F02. The DLS analysis revealed that AAV-5 in both the reference buffer and formulation F02 demonstrated a constant AAV-5 main peak with particles of approximately 34 nm before and after accelerated aging. However, AAV-5 in F02 demonstrated an increased overall scattering intensity already in the non-stressed AAV sample, while AAV-5 in the reference buffer showed a detectable increase in overall scattering intensity only after 4 weeks at 40°C once the thermal ramp reached approximately 65°C. The increase in overall scattering intensity indicates the formation of capsid oligomers or even bigger particles during accelerated aging.

After the addition of a thermal ramp, the DLS analysis demonstrated that the stability of AAV-5 in F02 was already reduced at *t* = 0 days and did not further decrease during the 4 weeks storage at 40°C.

The additional application of a thermal ramp during MADLS analysis of the stressed and non-stressed AAV-5 samples did not affect capsid concentration (data not shown). It is likely that only a relatively small proportion of capsids aggregated, and therefore, the sensitivity of the method was not sufficient to measure the incremental reduction of capsid concentration.

### Capsid protein unfolding

The conformational stability of capsid proteins was first analyzed by studying protein unfolding with DSC and two forms of DSF, the intrinsic (NanoDSF) and the extrinsic (eDSF), using the fluorescent dye SYPRO Orange (Fig. 1).

A comparative evaluation of the techniques’ consumption of virus material with the respective instruments, the possibility of high-throughput screening, and the amount of lab work associated with these techniques suggests that NanoDSF and eDSF are the most suitable methods for formulation development (Table 2).

Measuring protein unfolding of the non-stressed AAV-5 in the reference buffer using these three techniques revealed comparable midpoints of capsid protein unfolding (*T*_*m*_) at 90°C (data not shown). The onset of protein unfolding (*T*_ON_) differed only by 1°C between DSC (86°C) and NanoDSF (87°C).

Further evaluation of protein unfolding was performed with DSC and NanoDSF, as the analysis by eDSF using SYPRO Orange relied on the extrinsic fluorescent dye to bind to hydrophobic patches of proteins.

In order to evaluate the potential of NanoDSF and DSC to either predict or indicate AAV stability in different formulations, an accelerated aging study with AAV-2 and AAV-5 in up to seven different formulations was performed. The applied stress consisted of storage for 4 weeks at 40°C. The samples were analyzed before and after thermal stress application by NanoDSF and DSC.

**Fig 5:**
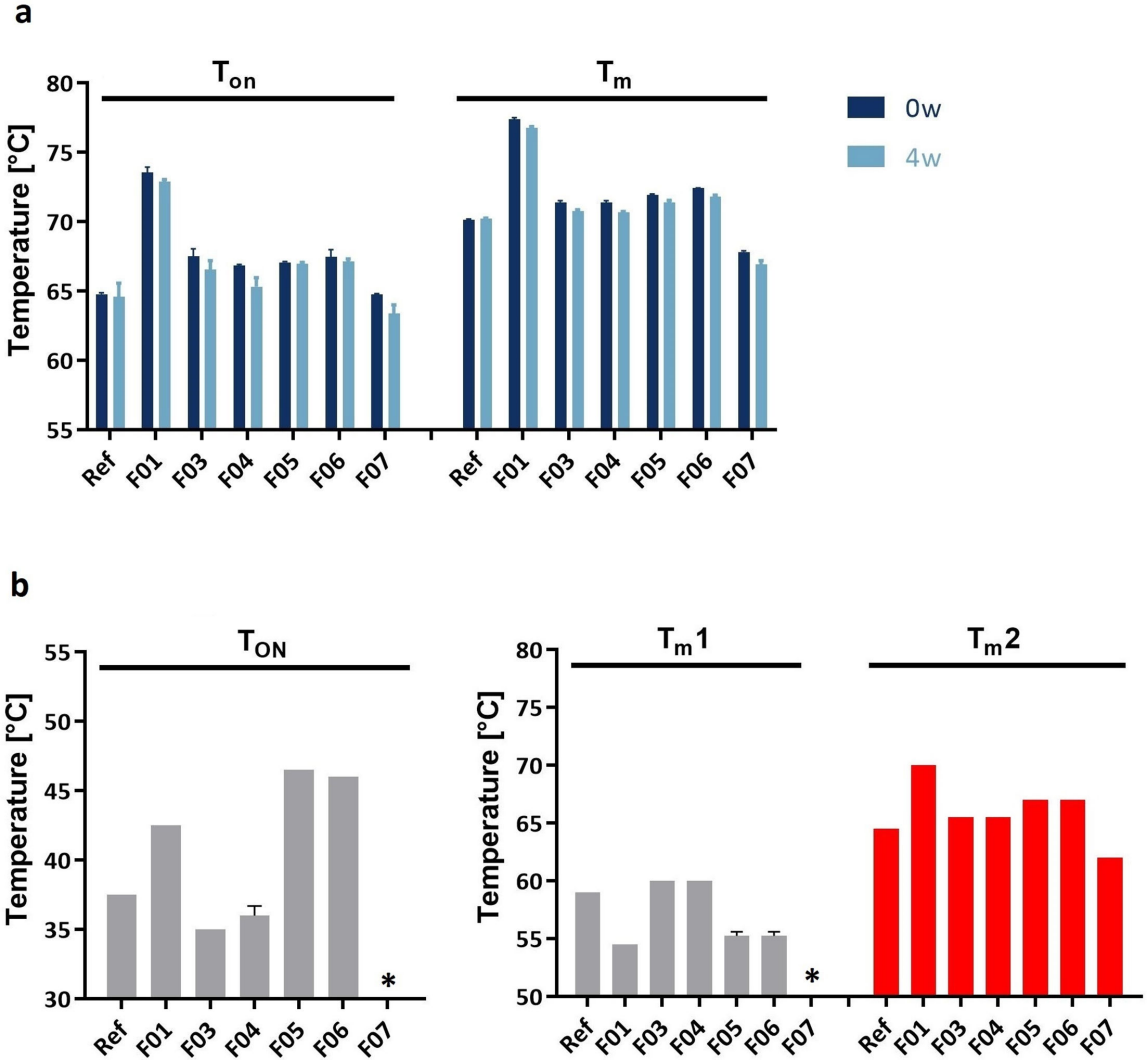
Capsid protein unfolding (a) and DNA release (**b**) of AAV-2 in reference buffer and seven other formulations (F01–F07) measured by NanoDSF and eDSF (SYBR Gold), respectively. (**a**) NanoDSF (*n* = 2) of AAV-2 before (*t* = 0 days) and after (*t* = 4 weeks) thermal stress at 40°C for 4 weeks. Depicted are the parameters *T*_ON_ and *T_m_*. (**b**) eDSF using SYBR Gold (*n* = 2) of AAV-2 before (*t* = 0 days) thermal stress at 40°C for 4 weeks. Depicted are the parameters *T*_ON_ and *T_m_*. Samples for which no value was obtained are marked with *.

**Fig 6:**
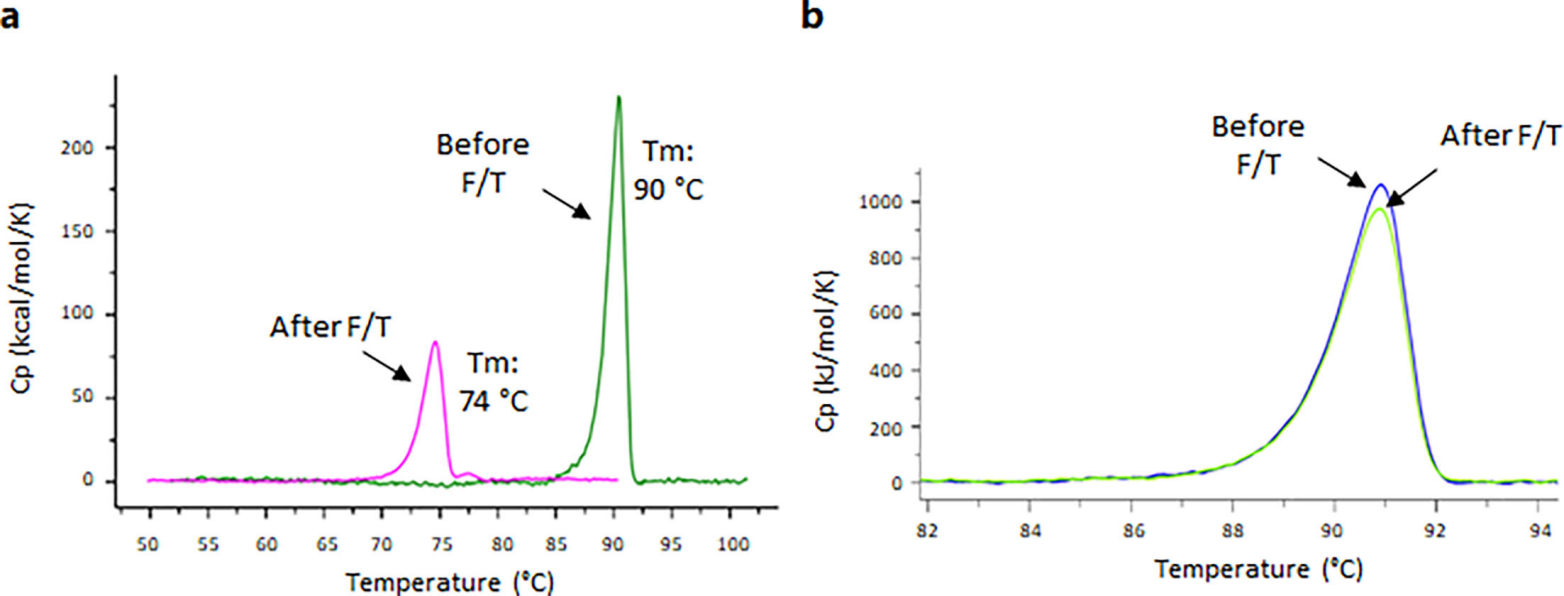
Thermogram as recorded by DSC of AAV-5 in reference buffer (**a**) and formulation F01 (**b**) before and after one freeze-thaw cycle.

For both analytical methods, only one unfolding transition was observable for non-stressed AAV-2 and AAV-5, with a *T*_*m*_ of the transition being characteristic for the serotypes (Fig. 5a and 6). No ssDNA peak was observed, which was previously reported for AAV of the same serotype and similar capsid titer in DSC (6). This absence can be attributed to a significantly lower content of full AAV-5 capsids in the samples analyzed in the present study.

The NanoDSF data obtained from AAV-2 analysis demonstrate no relevant impact of the accelerated aging condition for AAV in any of the investigated buffers (Fig. 5a). The unfolding of capsid protein in stressed and corresponding non-stressed samples differed in *T*_ON_ and *T*_*m*_ by a maximum of 1.3°C and 0.9°C, respectively. However, the analytical method revealed a pattern of formulations with high stabilizing (higher *T*_ON_ and *T*_*m*_) and less stabilizing effect for the non-stressed AAV samples, which was already observed by DSC (data not shown).

DSC analysis of AAV-5 samples subjected to different freeze-thaw cycles revealed the impact of the freezing and thawing process (Fig. 6). DSC analysis showed that AAV-5 in the reference buffer exhibited a significantly lower *T*_*m*_ after an additional freeze–thaw cycle was applied (Fig. 6a).

### Genome release from AAV capsids

Besides protein unfolding, another possibility to monitor capsid stability is to analyze the genome release from AAV capsids. In this study, the analytical technique eDSF with the fluorescent dye SYBR Gold was performed (Fig. 1). The dye binds to DNA and enhances fluorescence upon binding. Measuring the non-stressed AAV-2 in the seven different formulations by eDSF with SYBR Gold demonstrated a *T*_ON_ between 35°C and 47°C and two *T*_*m*_ values for the DNA ejection process. For all samples, *T*_*m*_1 was detected between 55°C and 60°C and *T*_*m*_2 between 62°C and 70°C (Fig. 5b). The analytical method revealed a pattern of high and low *T*_*m*_ values for the tested formulations, which differed between *T*_*m*_1 and *T*_*m*_2 (Fig. 5b) and are indicative of stabilizing effects of the different formulations.

### Capsid concentration and genome load

Several analytical methods allowing capsid quantification and measurement of the genome load were evaluated in this study (Fig. 10).

In order to determine the AAV capsid concentration, MADLS, ELISA (commonly used), SEC-MALS, and SEC-SLS (static light scattering) were performed. The results of all techniques matched the expected capsid concentrations very well (based on manufacturer information and previous SEC-MALS data as summarized in Table 3).

**TABLE 3:** Results of AAV capsid concentration analysis and genome load determination with different analytical methods^*a*^^,^^*b*^

Virus	Expected capsid titer (cp/mL)	Expected genome titer	Expected genome load (% full capsids)	Analytical method evaluated	Measured capsid titer		Determined genome load	
					Mean (cp/mL)	SD (%)	Mean (%)	SD (%)
AAV-5	3.1E + 14**	1.00E + 13	3.3	MADLS	4.30E + 14	n.a.	2.3***	n.a.
				SEC-MALS	2.00E + 14	0	3.9	0.1
				AUC	–	–	6	n.a.
AAV-2	6.35E + 12*	5.00E + 12	78.7	ELISA	6.30E + 12	5.03	79.0***	n.a.
				AEX-HPLC	–	–	91.1	0.1

^*a*^ *Value obtained by ELISA, which was performed by AAV manufacturer. **Value obtained by SEC-MALS, which was performed in a previous study by investigator. ***Calculated based on qPCR data.

^*b*^ “–” not measure, n.a. not applicable.

**Fig 7:**
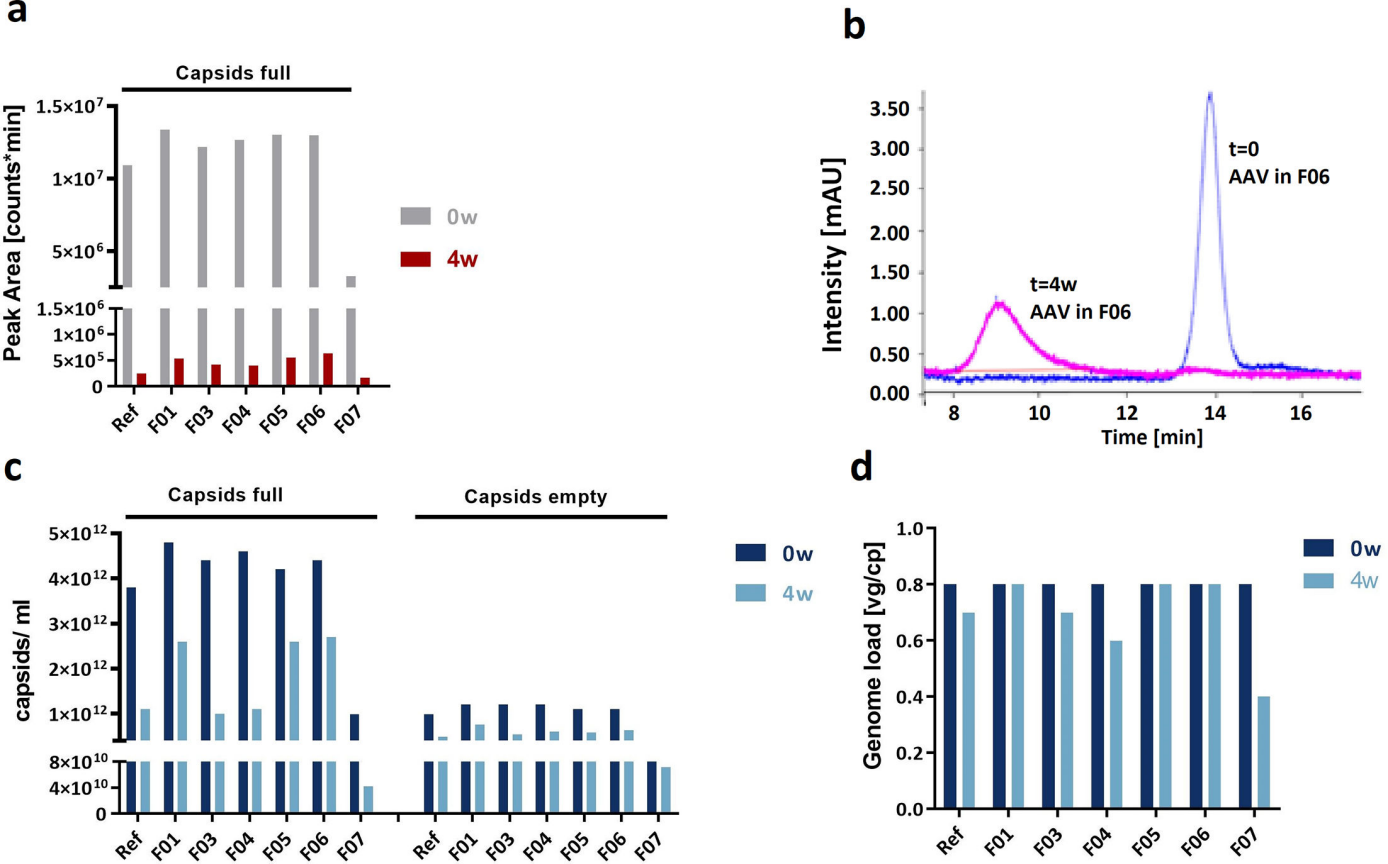
Amount of full capsids for AAV-2 in seven different buffers before (*t* = 0 days) and after (*t* = 4 weeks) storage at 40°C for 4 weeks measured by AEX-HPLC (**a and b**) and SEC-MALS (**c and d**). (**a**) Relative area for full capsids determined by UV detection, (**b**) chromatogram of AAV-2 in formulation F06 measured at 260 nm, (**c**) concentration of full and empty capsids determined by SEC-MALS (UV-MALS detection), and (**d**) genome load depicted as ratio of full/empty capsids determined by SEC-MALS (UV-MALS detection).

In order to evaluate the potential of SEC-MALS to indicate or predict AAV stability based on capsid quantification and genome load determination, the AAV-2 in reference buffer and formulations F01–F07 from the accelerated aging study were analyzed before and after 4 weeks of storage at 40°C. We observed a considerable decrease in full and empty capsids after thermal stress application and a pattern of formulations with high stabilizing effect (high amount of retained full capsids and high full/empty capsid ratio) and low stabilizing effect after 4 weeks (Fig. 7b and c).

Another possibility to determine genome load is provided by AEX-HPLC, which separates AAV capsids with different genome loads based on their net surface charge. AEX-HPLC of AAV-5 and AAV-2 allowed for the separation of empty and full capsids, but no peak corresponding to partially filled capsids was observed. Accordingly, the apparent genome load for AAV-2 determined by AEX-HPLC was higher than that estimated by combining ELISA and qPCR analysis.

In order to evaluate the stability-indicating and predictive potential of AEX-HPLC, the AAV-2 in seven formulations from the accelerated aging study was measured before and after 4 weeks of storage at 40°C. The fraction of full capsids differed slightly before stress application (Fig. 7a). After thermal stress application, viruses in all formulations showed a massive decrease in the number of full capsids (Fig. 7a) when judged by the particles eluting at the expected elution time. We observed a certain pattern of formulations with high and low amounts of retained capsids at the expected elution time. The fraction of particles with an elution behavior typically recorded for empty capsids increased, but at the same time, demonstrated a UV signal at 260 nm that was higher than the UV signal at 280 nm, indicating the presence of nucleic acids rather than proteins (data not shown).

In contrast to the techniques evaluated before, the analysis of AAV by AUC revealed a population of empty, full, as well as partially filled capsids. The analysis of AAV-5 resulted in 6% full capsids (UV 280 nm), which is only slightly higher compared to the results obtained by MADLS, SEC-SLS, and SEC-MALS (Table 3). The response factor, which is normally used to correct the values for 260 and 280 nm measurements in AUC, could not be determined, as there was no sample of 100% empty and 100% full capsids available.

**Fig 8:**
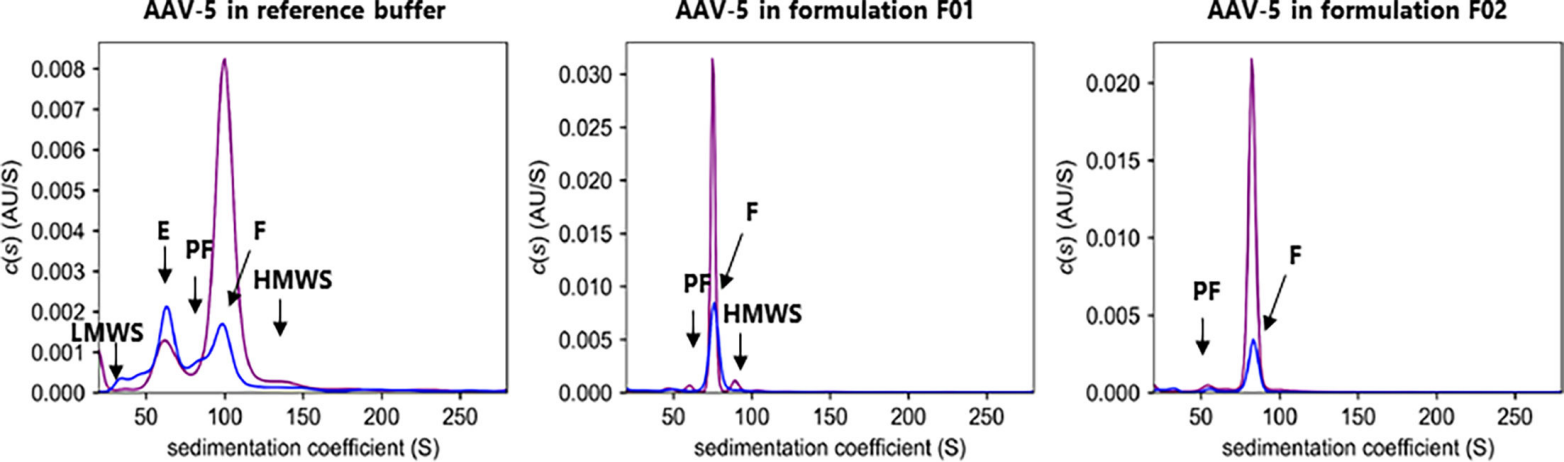
AUC results of AAV-5 in reference buffer and formulations F01 and F02 before (*t* = 0 days, purple) and after storage at 40°C for 6 weeks (*t* = 6 weeks, blue). Capsid description: E (empty), PF (partially filled), and F (full). Others: HMWS (high-molecular-weight species) and LMWS (low-molecular-weight species).

For further evaluation of AUC, the AAV-5 sample in reference buffer was compared with those in formulations F01 and F02 (Fig. 8). For non-stressed AAV-5 in all buffers, full capsids formed the main part of the detected particles, while empty capsids, partially filled capsids, and high and low molecular species only made up a minor part. The reference buffer was associated with poor properties, including the highest proportion of empty capsids and the highest impurity according to the width of the main peak.

The application of thermal stress (6 weeks at 40°C) decreased the amount of full capsids in all three buffers, with AAV-5 in the reference buffer showing the lowest amount of full capsids and a relatively high amount of empty capsids. This demonstrates that the reference buffer had the lowest stabilizing effect (Fig. 8).

In agreement with a previous publication (4), full AAV capsids in the reference buffer had a sedimentation coefficient of 100 S as determined by AUC. The sedimentation coefficient of AAV in F01 and F02 differed, although the data were corrected for the density and viscosity of the formulations.

Evaluating the virus material for measurements with the respective instruments, the possibility of high-throughput screening, and lab work associated with these techniques revealed that SEC-MALS and SEC-SLS were the best options for screening purposes during formulation development. AUC required the largest quantity of AAV material and lab work (since impurities made re-buffering necessary) and did not allow high throughput (Table 2).

### Virus infectivity

For a complete AAV stability analysis, the virus infectivity is an important attribute to monitor. For the replication-incompetent AAV, the technique laser force cytology (LFC) was evaluated, which allows a label-free microfluidic cell analysis on a single-cell level.

As a positive control, the replication-competent adenovirus (non-enveloped) and enveloped modified-vaccinia ankara virus (MVA) were used.

**Fig 9:**
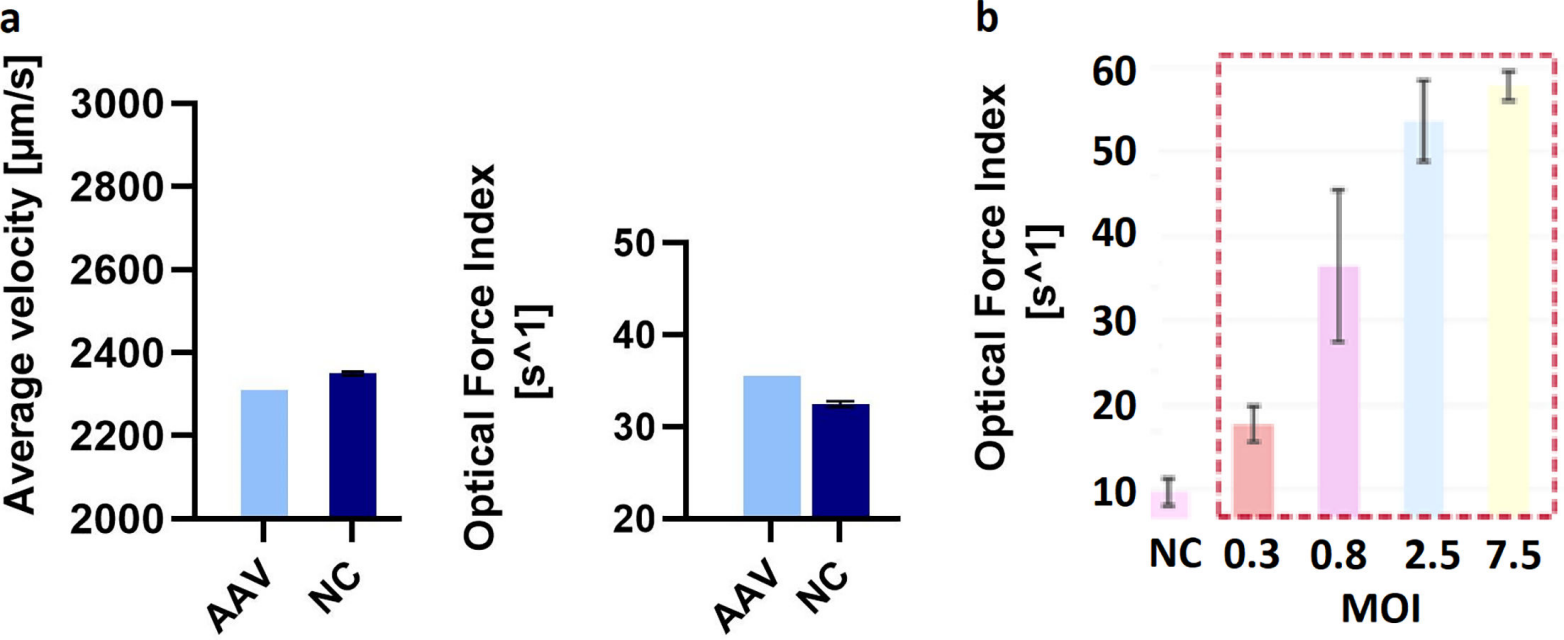
Laser force cytology results for AAV-2 (**a**) and adenovirus (**b**). (**a**) The average velocity (left) and optical force index for AAV-2-infected and non-infected (NC) HEK-293 cells. (**b**) The correlation of adenovirus amount used for infection (multiplicity of infection, MOI) and the LFC parameter optical force index.

Non-stressed AAV were analyzed at 5E + 12 vg/mL with LFC 3 days post-infection. For the parameters average velocity and optical force index, no relevant difference was observed for one AAV-infected sample compared to three uninfected controls (Fig. 9a). A separate experiment that included AAV concentration series (highest concentration being 5E + 12 vg/mL) analyzed 1, 2, and 3 days post-infection did not show a consistent correlation between any LFC parameter and AAV concentration (data not shown).

However, when the adenovirus was analyzed as positive control using LFC, with the optical force index serving as the decisive parameter, it was possible to distinguish very well between infected and non-infected cells. A good correlation was found with the amounts of virus used (Fig. 9b). A similar outcome was achieved with the enveloped MVA after accelerated aging when compared to results from TCID_50_ (data not shown).

## DISCUSSION

**Fig 10:**
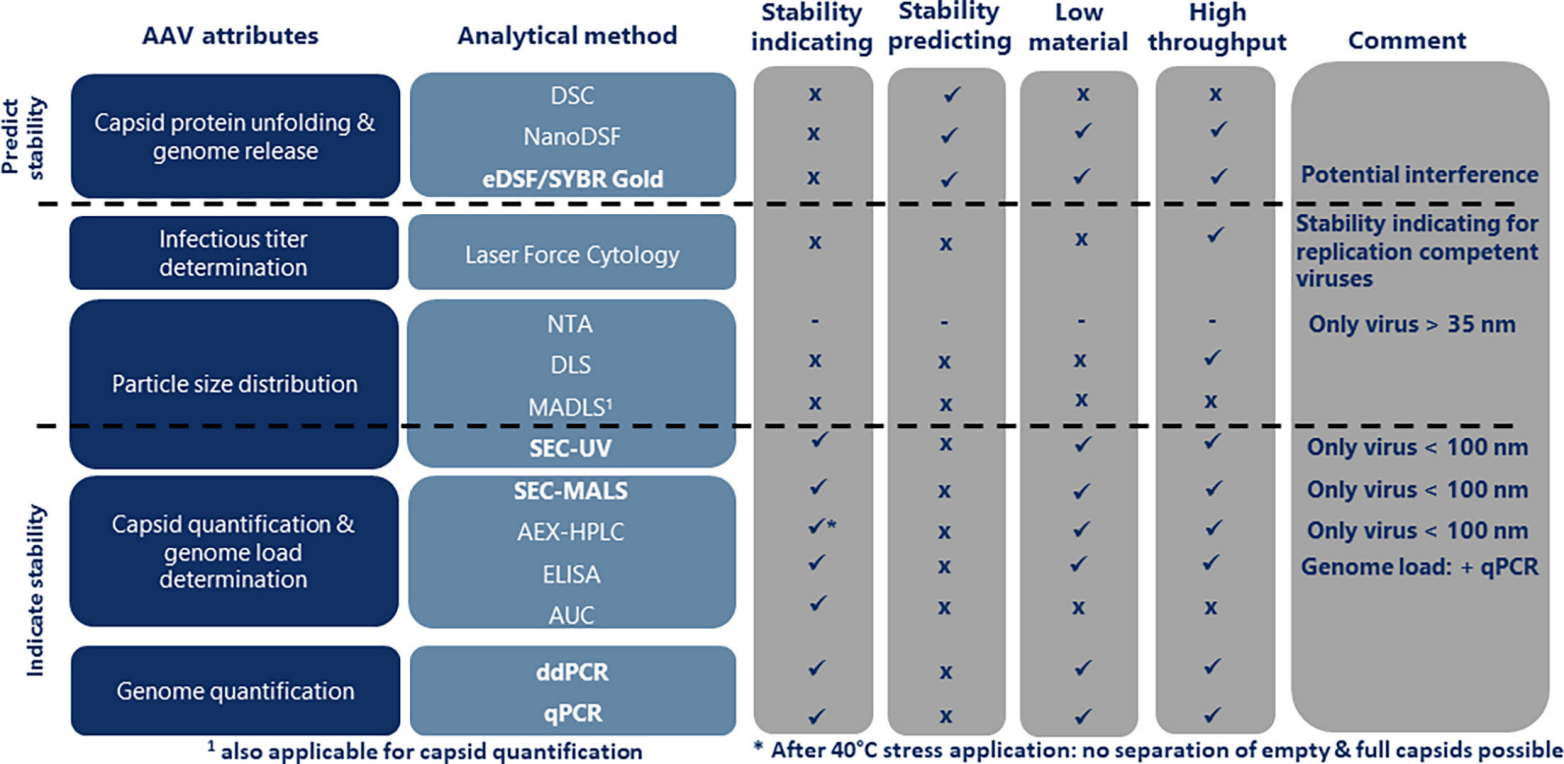
AAV attributes and analytical methods evaluated in this study for applicability and performance in AAV formulation development. Analytical methods in bold fulfilled the requirements for AAV formulation development.

AAVs are currently the most prominent viral vectors in gene therapy development, and optimal formulation conditions ensure their application success by increasing their resistance to various stress factors. A tailored formulation development includes monitoring capsid stability, particle interaction, and virus infectivity. Hence, a variety of analytical methods is required. In this study, we evaluated techniques for studying the most important virus attributes and analyzed whether these techniques have the potential to indicate AAV stability or predict stabilizing effects of formulations on these viruses (Fig. 10). Additionally, we determined whether these techniques allow for high-throughput screening and require only a low amount of virus material and lab work, which would be beneficial because applying these techniques during formulation development often requires measurements of a high sample number.

### Particle size distribution analyzed by DLS, MADLS, and SEC-UV

In particle size distribution analysis, DLS, MADLS, and SEC-UV are common techniques and were evaluated in this vector study.

SEC-UV and isothermal MADLS recorded for the non-stressed AAV-5 samples showed three particle populations, which could be assigned to (i) the main population predominantly comprising monomeric AAV-5, (ii) oligomers, and (iii) aggregates. The DLS measurement detected only two populations, indicating that DLS is less sensitive than SEC-UV and MADLS in this context. A quantitative analysis of particle populations by DLS is usually difficult due to the nonlinear relationship between particle diameter and light scattering intensity.

Nanoparticle tracking analysis (NTA), also a common technique for particle size distribution analysis, was considered to be evaluated as an orthogonal method to DLS, since it provides a number-weighted distribution, is very sensitive, and can distinguish different particle populations that are very similar in size. Furthermore, it records particle concentrations as well. Currently, however, there is no NTA technology/instrument available that is capable of analyzing AAV monomers with a diameter of approximately 25 nm and a small refractive index (1.45–1.5) (1). For bigger non-enveloped viruses, such as adenoviruses or enveloped viruses, however, NTA may be a suitable method.

None of the tested particle size analytical methods (DLS, SEC-UV, and MADLS) registered any difference between the non-stressed formulated AAV samples, indicating that they do not possess the potential to predict AAV-5 stability from untreated samples.

SEC-UV demonstrated the stability-indicating potential after accelerated aging, as additional aggregate formation for AAV-5 in the tested buffers was detected.

Combining DLS measurements with the application of a thermal ramp recorded minor differences in scattering intensities of the non-stressed AAV-5 samples. However, the early increase in overall scattering intensity detected for AAV-5 in formulation F02 at timepoint *t* = 0 (despite the constant radius of the main peak) indicates an early formation of oligomers or bigger particles. As scattering intensity relates to size to the power of six, the scattering signal is extremely sensitive to the presence of species of larger size.

Further research is necessary to confirm the effects and allow assertions about stability indication or prediction for DLS with thermal ramp application. The functionality enabling analysis of the temperature of the onset of scattering intensity could potentially be used for stability indication or prediction in formulation development.

In contrast, combining MADLS with the application of a thermal ramp did not affect capsid concentrations of any of the tested AAV-5 samples, indicating that this analytical method was not able to detect the formation of a relatively small fraction of oligomers and might be hidden within the assay variability.

Taken together, these facts increased our interest in using SEC-UV as a well-suited stability-indicating method for size distribution analysis in AAV formulation development. SEC-UV was also the only technique fulfilling the requirements for high throughput, low expenditure of lab work, and low virus material requirements. The latter could be further decreased by substituting the UV detector with a fluorescence detector.

### Capsid protein unfolding and genome release

DSC, NanoDSF, and eDSF monitor capsid protein unfolding based on different principles but showed very similar results regarding *T*_*m*_ and *T*_ON_ determination in our study, which were also in line with previous publications (2, 3, 8). For eDSF, a potential impact of the extrinsic fluorescent dye on the results of future formulation development could not be ruled out. Therefore, this technique using SYPRO Orange was less favorable when compared to NanoDSF and DSC.

Performing DSC with the stated instrument required a lot more virus material than NanoDSF and did not allow high-throughput screening, which raised our interest in NanoDSF for protein unfolding analysis in formulation development, with usually high sample numbers. However, it is important to mention that in previous NanoDSF measurements with proteins, we observed a strong interference of formulations containing tryptophan with the measurement of intrinsic protein fluorescence, since the main contribution of intrinsic fluorescence originates from aromatic amino acids such as tryptophan (data not shown).

In comparison to NanoDSF, DSC studies provide further information about the thermodynamic stability of capsid proteins due to thermodynamic parameters like the enthalpy of protein unfolding (5, 9). The enthalpy was not further analyzed within the course of this study; however, DSC may be an interesting tool for initial AAV characterization or final optimization of an AAV formulation. DSC may also detect DNA transitions and could be used as an indicator of transgene presence (5).

The analytical methods used to analyze capsid protein unfolding were additionally evaluated for their potential to indicate AAV stability after accelerated aging. Unlike HPLC techniques or AUC, DSC and NanoDSF were not able to detect a relevant impact of thermal stress from accelerated aging, demonstrating that they were not stability indicating when subjected to thermal stress.

However, when AAV-5 samples were subjected to an additional cycle of freeze and thaw (at −20°C), the DSC analysis demonstrated differences between AAV-5 stability in the tested buffers. Cryogenic separation of PBS buffer compounds and related pH shifts of PBS upon freezing may destabilize therapeutic molecules (10). This might explain the *T*_m_ shift detected by DSC for the AAV-5 in the tested reference buffer (PBS with 0.001% poloxamer 188). Additionally, it is reported that capsid protein unfolding under thermal stress is also strongly affected by the buffer system, with PBS demonstrating one of the lowest stabilizing effects on AAV (3). In our study, alternative formulations had considerably higher stabilizing effects on the AAV serotypes regarding thermal unfolding. Based on our findings and previous reports (6), DSC and NanoDSF may be useful tools to quickly screen buffers for their stabilizing potential on AAV.

Interestingly, both methods, DSC and NanoDSF, detected differences between AAV in distinct buffers without stress model application and demonstrated formulations with high and lower stabilizing effects as concluded from unfolding transition temperatures. Since SEC-MALS demonstrated highly similar profiles of more or less stabilizing formulations accelerated aging at 40°C for 4 weeks, the observations indicate that NanoDSF and DSC may have meaningful potential to predict the formulation’s power of stabilizing AAV. While the detected differences were rather small for the stable AAV-5, they were more prominent for the less stable AAV-2.

Compared to the SEC-MALS results and the outcome of NanoDSF regarding protein unfolding, eDSF using SYBR Gold to track the genome release from AAV capsids demonstrated the same pattern of differently stabilizing formulations for *T*_*m*_2 (eDSF) without the application of stress. This indicates that this method may also have predictive power regarding virus stability.

The eDSF profiles of formulations with low and higher *T*_ON_ and *T*_*m*_1 differed from the formulation profile obtained by SEC-MALS for full capsids after stress model application and by NanoDSF at *t* = 0 weeks. This indicates that early genome release is an important attribute to analyze when evaluating AAV stability. Therefore, eDSF using SYBR Gold provided crucial additional information about AAV stability by determining *T*_ON_ and *T*_*m*_ of genome ejection.

### Capsid concentration and genome load

In the third part of the study, we evaluated analytical methods that determine AAV concentration and genome load, as partially filled capsids and empty capsids may be responsible for decreased dosage and increased risk of immunogenicity (11, 12).

In contrast to MADLS, ELISA and SE-HPLC-based methods allowed capsid concentration measurement with high throughput and were additionally very precise in capsid concentration determination, which makes them very interesting for screening purposes during formulation development. However, ELISA is very cost-intensive when purchasing coated plates, requires more lab work than HPLC methods, and an additional assay, such as qPCR or ddPCR, is necessary to obtain information about the AAV genome load. SEC-MALS, on the other hand, combines capsid concentration measurement with genome load determination and particle size distribution analysis within one analytical step.

Based on the evaluation of different analytical methods for genome load determination within this study, SEC-MALS became the most interesting for formulation screening, since it was stability indicating, required less AAV material than AUC, did not depend on viscosity and density correction like AUC, and was not affected by the modified net charge of AAV capsids with thermal stress application as observed in AEX-HPLC. For a complete analysis, including the detection of partially filled AAV particles, AUC analysis could be added in the final steps of formulation development and applied as an orthogonal method for the best-performing formulations from previous screening steps analyzed by SEC-MALS.

The AEX-HPLC result from AAV-2 analysis differed slightly from the expected value based on ELISA measurements by the AAV vendor. This may result from the missing correction for the response factor of DNA and protein at 260 and 280 nm, respectively, in AEX-HPLC measurements, because the response factors could not be determined at that time. However, in formulation development, only relative comparisons between different formulations of the same virus sample are performed, and a correction for response factors, therefore, is not necessarily required.

Taken together, both HPLC techniques (SE- and AEX-HPLC) and AUC were stability indicating, and AEX-HPLC additionally demonstrated small differences between the formulated AAV-2 samples even before thermal stress application. These differences predicted the stabilizing properties of different formulations that were confirmed by SEC-MALS/SEC-UV measurements after 4 weeks at 40°C. AEX-HPLC may have predictive potential, which should be further investigated in future studies.

### Virus infectivity

For a complete AAV stability study, AAV infectivity should be considered. As functionality assays, such as reporter gene assays, are already established for rapid quality control assessments, those are not addressed in this study.

Since AAVs are replication incompetent without helper viruses, we tested LFC as an alternative to traditional assays such as TCID50. We evaluated LFC using AAV-2, and as a positive control, we chose the replication-competent adenovirus (non-enveloped) and the enveloped MVA. While LFC was only able to detect slight differences between non-infected HEK-293 cells and a high concentration of AAV-infected population, it was not able to determine the infectious AAV titer in this study, even when using the unconcentrated virus correlating to a MOI of 1.26E + 06. For replication-competent viruses such as adenovirus and MVA, however, LFC has been shown to be stability indicating. LFC is a considerably attractive alternative to traditional assays such as TCID50 or virus titration with subsequent hexon staining (13, 14).

### Conclusion

For each relevant AAV attribute, at least one suitable method could be identified that fulfilled the requirements for AAV characterization and stability analysis during formulation development (except for infectious titer determination). These were defined to indicate or predict the stability of AAV vectors (Fig. 10). Testing the functionality of the vectors was outside the scope of this study but should be added to any formulation development study at a certain stage to confirm the viability of formulations considered for further development. The DSC method, in particular, made it possible to demonstrate the unsuitability of PBS for stabilizing AAVs against freeze/thaw-induced stress and thus demonstrated the importance of formulation development in this area.

## MATERIALS AND METHODS

### Formulated virus samples and accelerated aging studies

AAV-2 and AAV-5 (Sirion Biotech GmbH) in reference buffer (2.7 mM KCl, 1.5 mM KH_2_PO_4_, 137.9 mM NaCl, 8.1 mM Na_2_HPO_4_, and 0.001% poloxamer 188, pH 7.4) were used as reference. AAV-5 samples with only 3%–6% full capsids were used as control reference samples representing AAV-5 with mainly empty capsids. Furthermore, seven different formulations (F01–F07, Table 4) were used in this study for comparison. In the accelerated aging study (ICH-Q1 compliant), samples were stored at 40°C ± 2°C at controlled humidity of 75% ± 5%. Adenovirus (serotype 5) was formulated in five different formulations (F08–F12, Table 4) and stored at 5°C ± 3°C for 4 years.

**TABLE 4:** Formulation buffers for AAV and Ad

	Formulations for AAV							Formulations for Ad				
Excipient	F01	F02	F03	F04	F05	F06	F07	F08	F09	F10	F11	F12
Alanine (g/L)										10		
Arginine (g/L)				6.5	21.3				15			
Glutamic acid (g/L)								10				
Glycine (g/L)				2.8	8.5							
Hepes (g/L)											2.38	2.38
Histidine (g/L)		3.1		1.6	3.1		3.1	25	30	3		
Lysine-HCl (g/L)										31.24		
Methionine (g/L)				1.5	1.1			1.5	1.5	1.5		
MgCl_2_·6H_2_O (g/L)		0.4	0.4	0.2	0.3		0.4	0.41	0.41	0.41		0.41
NaCl (g/L)		8.8		8.8			8.8					
PBS (g/L)						Ready-to-use						
Poloxamer 188 (g/L)	0.01	0.01	0.01	0.01	0.01	0.01	0.01					
Sucrose (g/L)		30		15	42.6		30	40	40	40	40	40
Trisodium citrate·2H_2_O (g/L)	26		26	3.7								
Tris (g/L)	1.2	2.4	1.2	2.4			2.4					
Tryptophan (g/L)									2			
pH	7.4	8	7.4	8.5	7.4	7.4	8	7.4	7.4	7.4	8	7.4

### eDSF

Formulated AAV-2 in 10× SYBR Gold (Thermo Fisher Scientific, S11494) was measured at a concentration of 8.0E + 11 vg/mL with Thermal Cycler CFX96 (BioRad) and a thermal ramp of 28°C–86°C, 0.5°C/min, and 90 s hold. AAV-2 dilution to the target concentration was performed with the corresponding buffer. *T*_*m*_ was determined by the Bio-Rad CFX manager software.

### NanoDSF

Formulated AAV-2 was measured at 3.0E + 12 cp/mL by the Prometheus NT.Plex (NanoTemper Technologies GmbH) with a thermal ramp of 30°C–90°C, 1.5°C/min. The parameters *T*_*m*_ and *T*_on_ were determined by PR.Stability Analysis software.

### DSC

For the analysis, 325 µL of each formulated AAV-5 sample and its matching buffer were analyzed in a sealed 96-well plate with the automated MicroCal PEAQ DSC (Malvern Panalytical). Thermal scans were performed in the range from 4°C to 130°C at a scan rate of 60°C /h, and intensive cleaning steps between the samples were conducted. Data were analyzed with dedicated PEAQ DSC Analysis software (Malvern Panalytical).

### AEX-HPLC

AEX-HPLC was performed on a Dionex Ultimate 3000 instrument (Thermo Fisher Scientific) at 30°C with a Protein-Pak Hi Res Q column (5 µm, 4.6 × 100 mm, Waters GmbH) and a salt gradient (buffer A: 0.07 M bis-tris propane, pH 9.0, buffer B: 0.07 M bis-tris propane and 1 M Tetraammonium chloride, pH 9.0). Fluorescence was measured at (ex) 280 nm and (em) 350 nm. The amount of full and empty capsids was determined by manual peak integration in Chromeleon Software.

### SEC-UV and SEC-SLS with AAV-5

In SE-HPLC, AAV was separated on a Superose 6 column (GE Healthcare, 29091596) using a Dionex Ultimate 3000 system (Thermo Fisher Scientific). The samples were injected non-diluted (10 µL) at a concentration of 4.6E + 13 cp/mL. The measurements were performed using PBS (Life Technologies, 14190250) as mobile phase buffer at an isocratic flow of 0.7 mL/min at 30°C ± 2°C. Data were recorded at 280 and 220 nm by a UV detector (SEC-UV) and for MALS-SLS, additionally by an OMNISEC system (Malvern Panalytical) consisting of a refractive index, UV/Vis-photodiode array detection, and SLS detector, comprising two observation angles: right angle (90°) and low angle (7°) (SEC-SLS). Data analysis was performed with Chromeleon Software.

### SEC-MALS with AAV-2

In contrast to the information stated for AAV-5, the AAV-2 (35 µL of 8E + 11 cp/mL) was separated on an Analytical Column (WTC-050S5) with a flow rate of the mobile phase of 0.3 mL/min at 25°C. Here, a pre-column and the UV and MALS detector from Wyatt Technology were used.

### DLS and MADLS

AAV samples in concentration ranges from 5E + 12 to 1.5E + 13 cp/mL were loaded into quartz cuvettes (ZEN2112 Malvern Panalytical) at 20–50 µL. The measurements were conducted on Zetasizer DLS Ultra (Malvern Panalytical). For thermal ramp experiments, AAV samples were heated up at 0.5°C/min. Size measurements were performed in the temperature range from 25°C to 80°C at 1°C increments, and backscatter detection and particle concentration measurements were taken every 5°C. Particle size distribution and concentration analysis were performed using ZS Xplorer software (Malvern Panalytical). *T*_ON_ temperatures were deduced from thermal ramp experiments by a dedicated statistical method following the upturn of the derived count rate signal.

### AUC

Sedimentation velocity analysis was carried out in single replicate measurements at 20°C and 15,000 rpm using an Optima AUC (Beckman Coulter) in an analytical ultracentrifuge equipped with an AN-Ti50 rotor or AN-Ti60 and absorbance optics. UV absorbance detection was performed at 280 nm. The distribution of sedimentation coefficients and molecular weight high-throughput screening was determined mathematically using the c(s) model. Data analysis was performed with the program Sedfit. The noise and baseline of the obtained data, as well as the sample meniscus and the frictional ratio, were fitted during sample analysis, while the cell bottom distance value was fixed. Afterward, the obtained sedimentation distribution was plotted using the GUSSI tool.

### Cell culture

HEK-293 cells were cultivated in DMEM (Gibco, 21969035) with 10% FBS (Gibco, S0615), 1% 200 mM L-Alanine-L-Glutamine (Gibco, G8541), and 1% 100 U/mL Penicillin/Streptomycin (Sigma-Aldrich, P0781).

### Antibody-based infectivity assay with subsequent hexon staining

In a 24-well plate, 500 µL containing 2.5 × 10^5^ HEK-293 cells were seeded per well. After 3 h of cell attachment, serial dilutions of samples were prepared in cell culture medium. A volume of 50 µL of the respective dilution was added per well. Non-infected HEK-293 cells were used as a negative control. After infection, cells were incubated for 42 ± 2 h at 37°C (+5% CO_2_) and subsequently fixed with methanol. Immunostaining was performed stepwise by incubation with the primary anti-hexon protein antibody (Santa Cruz Biotechnology, sc-51746), the secondary horseradish peroxidase (HRP)-conjugated anti-mouse antibody (Cell Signaling Technology, 7076S), and with the Vector NovaRED Substrate Kit (Biozol, SK-4800) to enable an HRP enzymatic reaction. The quantification of infected cells was performed by counting stained, brown-colored cells under a light microscope (CKX53, Olympus). Up to five visual fields per well were counted. Each stained cell was considered as one infective viral particle in order to calculate the infective units per milliliter (IU/mL).

### Infectivity assay based on LFC

With LFC, phenotypic changes in cell biochemistry and morphology (e.g., cytoskeletal changes) typically observed upon virus infection are recorded. These phenotypic changes can give rise to detectable differences in optical force, deformability, and other multi-variate data parameters quantified by Radiance (LumyCyte).

In a 24-well plate, 50 µL containing 0.2 × 10^6^ HEK-293 cells were seeded per well. After 3 h of cell attachment, adenovirus samples were diluted to a final concentration of 3.0E + 07-1.1E + 06 IU/mL. A volume of 50 µL of the respective dilution was added per well. In the case of AAV analysis, 2.5E + 11 vg were used for infection, which correlates to an MOI of 1.26E + 06. Non-infected HEK-293 cells were used as a negative control (Ad_NC). After 48 h (Ad5) or 72 h at 37°C (+5% CO_2_) (AAV-2), the supernatant (with detached cells) was collected and temporarily stored in a separate 96-well plate. Adherent cells were detached using TrypLE and resuspended in the separately stored supernatant. The solution was diluted 1:2 with LumaCyte stabilization fluid, and cells were counted to confirm a concentration between 0.3 and 0.6 × 10^6^ cells/mL. After centrifugation (5 min at 200 *g*), the supernatant was discarded, and the cell pellet was resuspended in a minimum of LumaCyte stabilization fluid + 0.5% paraformaldehyde. Afterward, samples were transferred to LumaCyte 96-well plates for analysis with the Radiance instrument.

For data analysis, including the definition of reliable stability-indicating parameters, LumaCyte’s ReportR software was used.

## AFTER EPUB

*[This article was published on 30 June 2025 with an error in the author byline. The byline was corrected in the current version, posted on 8 July 2025.]*
